# The effectiveness of prehospital hypertonic saline for hypotensive trauma patients: a systematic review and meta-analysis

**DOI:** 10.1186/s12873-017-0146-1

**Published:** 2017-11-28

**Authors:** I. E. Blanchard, A. Ahmad, K. L. Tang, P. E. Ronksley, D. Lorenzetti, G. Lazarenko, E. S. Lang, C. J. Doig, H. T. Stelfox

**Affiliations:** 1 Alberta Health Services, Emergency Medical Services, Calgary, Alberta Canada; 2 University of Calgary, Cumming School of Medicine, Department of Critical Care, Calgary, Alberta Canada; 3 University of Calgary, Cumming School of Medicine, Department of Community Health Sciences, Calgary, Alberta Canada; 4 University of Calgary, Cumming School of Medicine, Department of Medicine, Calgary, Alberta Canada; 5 University of Calgary, Cumming School of Medicine, Department of Emergency Medicine, Calgary, Alberta Canada; 6 O’Brien Institute for Public Health, University of Calgary, Calgary, Alberta Canada

**Keywords:** Emergency medical services, Allied health personnel, Fluid therapy, Hypotension

## Abstract

**Background:**

The optimal prehospital fluid for the treatment of hypotension is unknown. Hypertonic fluids may increase circulatory volume and mute the pro-inflammatory response of the body to injury and illness. The purpose of this systematic review is to determine whether in patients presenting with hypotension in the prehospital setting (population), the administration of hypertonic saline (intervention), compared to an isotonic fluid (control), improves survival to hospital discharge (outcome).

**Methods:**

Searches were conducted in Medline, Embase, CINAHL, and CENTRAL from the date of database inception to November, 2016, and included all languages. Two reviewers independently selected randomized control trials of hypotensive human participants administered hypertonic saline in the prehospital setting. The comparison was isotonic fluid, which included normal saline, and near isotonic fluids such as Ringer’s Lactate. Assessment of study quality was done using the Cochrane Collaborations’ risk of bias tool and a fixed effect meta-analysis was conducted to determine the pooled relative risk of survival to hospital discharge. Secondary outcomes were reported for fluid requirements, multi-organ failure, adverse events, length of hospital stay, long term survival and disability.

**Results:**

Of the 1160 non-duplicate citations screened, thirty-eight articles underwent full-text review, and five trials were included in the systematic review. All studies administered a fixed 250 ml dose of 7.5% hypertonic saline, except one that administered 300 ml. Two studies used normal saline, two Ringer’s Lactate, and one Ringer’s Acetate as control. Routine care co-interventions included isotonic fluids and colloids. Five studies were included in the meta-analysis (*n* = 1162 injured patients) with minimal statistical heterogeneity (*I* ^*2*^ *=* 0%)*.* The pooled relative risk of survival to hospital discharge with hypertonic saline was 1.02 times that of patients who received isotonic fluids (95% Confidence Interval: 0.95, 1.10). There were no consistent statistically significant differences in secondary outcomes.

**Conclusions:**

There was no significant difference in important clinical outcomes for hypotensive injured patients administered hypertonic saline compared to isotonic fluid in the prehospital setting. Hypertonic saline cannot be recommended for use in prehospital clinical practice for the management of hypotensive injured patients based on the available data.

PROSPERO registration # CRD42016053385.

## Background

The Emergency Medical Services (EMS) system is the “safety net” of health care responding to those critically injured or ill. Paramedics are trained to provide transport and emergency treatment on-scene and enroute to more definitive care. One such intervention is intravenous fluid therapy for patients with intravascular volume depletion. However, the optimal fluid management strategy for such patients is unknown [1–3]. Some reasons for this knowledge gap include differences in conditions giving rise to hypotension, timing of fluid therapy, comorbidities, fluid type, volume and rate of infusion. A common prehospital approach is to provide boluses of isotonic or near isotonic fluid (i.e., normal saline or Ringer’s Lactate) to maintain a target systolic blood pressure between 80 and 90 mmHg [2, 4].

### Importance

Hypertonic saline, whose composition of solutes is higher than that of the human body, has been hypothesized to exert a dual physiological role of increasing circulatory volume with minimal volumes of fluid, and muting the pro-inflammatory response of the body to injury and illness [1, 5]. It is this dual role that has led to the hypothesis that in hypotensive states, hypertonic saline may be superior to isotonic fluids in altering the causal pathway of low blood pressure resulting in tissue injury, leading to organ failure, which leads to further tissue injury and organ failure, and eventually death [1, 2, 6–8].

Hypertonic saline therapy is potentially appealing for prehospital care when compared to an isotonic fluid as it may allow infusion of lower fluid volumes, which when combined with permissive hypotension would decrease the amount of isotonic fluid administered prehospital. This strategy would align with the principles of damage control resuscitation that advocates for the judicious use of isotonic fluid, and the principles of targeted resuscitation; where clinical endpoints rather than fixed doses guide the volume of fluid infused [9, 10]. Operationally, the use of hypertonic saline would require only a small investment to add to existing fluid resuscitation protocols and may be particularly useful for teams operating in austere and remote conditions (e.g., tactical or wilderness EMS, etc.) where there are limitations to equipment availability and the time to definitive care is long.

In spite of promising animal and pre-clinical data, a systematic review and meta-analysis published in 2009 by the Cochrane Collaboration that included prehospital and hospital based studies of patients presenting with hypovolemia did not reach any definitive clinical conclusions on the impact of hypertonic saline [7]. They report a relative risk of mortality at hospital discharge for traumatically injured patients of 0.84 (95% CI 0.69,1.04) using a fixed effect model. These findings also align with another meta-analysis conducted in 2014 that included traumatically injured patients that presented with hypovolemia [11]. Like the Cochrane review, this study included hospital and prehospital administration of hypertonic saline. They report a pooled relative risk of mortality at hospital discharge using a fixed effect model of 0.96 (95% CI 0.82, 1.14).

The systematic review and meta-analysis reported here adds to existing knowledge by restricting study inclusion to prehospital fluid therapy, while being inclusive of all conditions that may give rise to hypotension.

### Goals of this investigation

To support evidence based prehospital clinical guideline development, the following question was asked: in patients presenting with hypotension in the prehospital setting (population), does the administration of hypertonic saline (intervention), compared to isotonic fluid (control), change survival to hospital discharge (outcome)? Secondary outcomes included longer-term survival, vital signs, fluid/blood requirements, Multiple Organ Dysfunction Score (MODS), length of hospital stay, disability and neurological outcome scales, and adverse events.

## Methods

This systematic review was conducted following a pre-specified study protocol registered with the International Prospective Register of Systematic Reviews (PROSPERO) (registration number CRD42016053385) and in accordance with the Preferred Reporting Items for Systematic reviews and Meta-analysis (PRISMA) [12].

### Search strategy

Searches were conducted in four electronic databases without language restriction, including Medline (1946 to Nov., 2016), Embase (1974 to Nov., 2016), CINAHL (1937 to Nov., 2016) and CENTRAL (Cochrane Central Registry of Controlled Trials – 1991 to Nov., 2016).

The search strategy comprised two concepts: prehospital setting and intervention. Different synonyms for each concept were identified from previous articles [13]. The Boolean operator “OR” was used to combine keywords and controlled vocabulary (e.g., medical subject headings - MeSH, etc.). The yield from each concept search was then combined by the Boolean operator “AND”. Two filters were subsequently applied to the combined results, the first filter restricted studies to randomized controlled trials (RCTs) and the second to human studies through the use of an animal study filter [7, 14, 15]. No RCT or human/animal filter was used for CENTRAL, and no animal study filters were identified for CINAHL. Given the existence of multiple RCTs found in pre-searches, and the low likelihood of finding a clinical trial in the grey literature, no grey literature searching was conducted. In addition, due to the heterogeneous nature of the conditions that may be amenable to receiving hypertonic saline in the prehospital environment, searching all EMS, emergency medicine, critical care, trauma, cardiac, and neurological conferences was beyond the scope of the resources available for this review.

### Inclusion and exclusion

There were six criteria specified a priori for inclusion and exclusion of studies in this systematic review:

1. Patients: both adult and pediatric populations were included.
2. Hypotension: defined as a systolic blood pressure of less than 100 mmHg or suspicion of the development of a hypotensive state. This conservative definition was used to ensure all relevant studies related to hypotension were captured. Mean arterial pressure was not an inclusion criteria since all studies identified used systolic blood pressure. All potential etiologies were included.
3. Prehospital setting: defined as treatment by an organized system of response, often referred to as EMS, prior to arrival at hospital.
4. Administration of hypertonic saline: hypertonic fluid was defined as any fluid that has a sodium chloride concentration greater than 0.9% (normal saline). The administration of a colloid in conjunction with hypertonic saline may have different clinical effects [2, 16, 17] and therefore studies were restricted to those that did not include a colloid as part of the RCT intervention [7, 8]. Studies where a colloid was considered part of routine care (co-intervention) and available to both intervention and control groups were included.
5. Control fluid: limited to isotonic or near isotonic fluids (e.g., normal saline, Ringer’s Lactate, etc.) [1, 7].
6. RCT study design: the Cochrane Collaboration’s definition of an RCT was adapted to this context and defined as patients who are prospectively assigned to receive hypertonic saline or isotonic intravenous fluid as described above [14]. Studies in which the allocation was not random and those in which it was quasi-random were not included.

### Identification of articles for eligibility

All candidate citation titles and abstracts were reviewed independently by two authors (IEB and AA) unblinded to author and other publication information. Disagreements between the two reviewers were resolved by consensus, and it was not necessary to use a third author (HTS) for adjudication.

Citations meeting inclusion and those in which any relevant inclusions were not clear were retained for full text review. Retained studies also underwent independent and unblinded full text review by the same authors, inclusion and exclusion criteria applied again, disagreements handled in the same manner, and a kappa score calculated for inter-rater agreement. Given the presence of multiple publications emanating from one study, particular attention was paid to ensure no duplicate data were included. In all cases, the publication outlining the main results of the study was used. Two reviewers (IEB and AA) hand searched bibliographies of articles that met inclusion criteria on full text review for studies that may not have been identified in the database searches.

### Data extraction

A data extraction form was developed prior to study selection, pilot tested on one of the included studies and refined accordingly as other studies were abstracted. This form was used to collect information on four constructs: population, intervention and control, outcome, and quality criteria. Data abstraction was performed by two authors (IEB and AA) with all data independently verified by each author, and discrepancies resolved by consensus. When data were not clear, the reviewers interpreted the data together and came to a consensus. Unclear data or areas where assumptions had to be made are highlighted.

### Risk of bias

A component approach was used to assess the risk of bias in included studies [12, 18]. The Cochrane Collaboration’s tool for assessing risk of bias was used as a broad framework for this assessment [14]. Three empirically demonstrated quality domains were the primary focus of the quality assessment, and included concealed allocation, blinded outcome assessors, and intention to treat analysis [12]. Other domains relating to quality were reported if they were considered to be relevant on review of the included studies. Risk of bias was assessed independently by two reviewers (IEB and AA), with consensus achieved through discussion as required. Bias was rated as low, high, or unclear for each component as per the Cochrane Handbook guidelines [14].

### Data synthesis and analysis

A PRISMA flowchart reported the number of included and excluded studies [12]. A summary of the essential characteristics of these studies are provided [18].

The primary outcome of interest was survival to hospital discharge, which was assessed in a two stage process. The first stage was the reporting of relative risks (RR) for survival from each individual study. The second stage was creating a weighted average RR for survival from all studies with a 95% confidence interval. The heterogeneity between studies was assessed using Cochran’s Q and *I* ^2^ statistics [12, 18]. Based on the results of these tests (Cochran’s Q *p* > 0.10, and *I* ^2^ < 40%), a fixed effect model was used and we assumed that any treatment effect was similar across studies [14, 18]. The fixed effect model was specified using the methods of Mantel and Haenszel [18, 19]. A sensitivity analysis was performed by re-running the analysis and excluding select studies (e.g., low quality, high statistical weight, etc.), to determine their effect on pooled results.

Secondary outcomes included long-term survival, vital signs, fluid/blood requirements, multiple organ dysfunction syndrome, length of hospital stay, disability and neurological outcome scales, and adverse events. These outcomes were described qualitatively. The most common unit of measure was used and transformations were performed as required (e.g., median to mean, interquartile range to standard deviation, etc.) [20].

Small study effect was assessed using a funnel plot to visually scan for asymmetry, and Egger’s and Begg tests as statistical analogies of the funnel plot [18]. All analyses were conducted using STATA (Version 11.2, College Station, Texas).

## Results

### Identification of studies

**Fig. 1:**
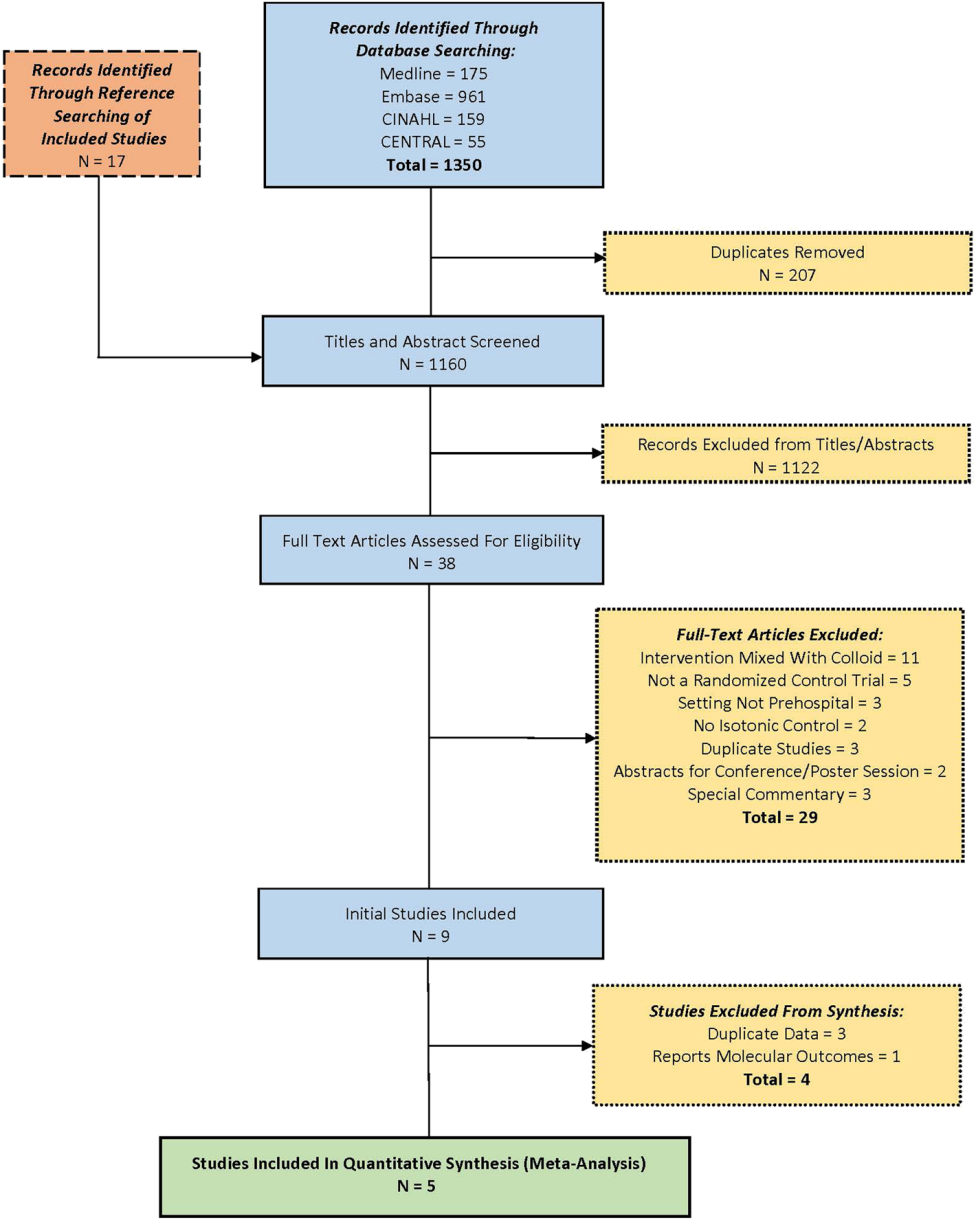
Review Inclusion

A total of 1350 studies were retrieved from the electronic database searches with 1160 studies undergoing title and abstract review (Fig. 1). Thirty eight studies were included for full text review. Nine studies were subsequently included from full text review (Kappa = 0.737), with all discrepancies resolved by consensus [16, 21–28]. Three of the included studies were found to report duplicate data from that presented in the main report of a trial and were excluded [26–28]. One further study was found to be a subgroup analysis associated with one of the included trials and did not provide outcomes aligning with the a priori methods, and was therefore excluded [16]. Of the five included studies, a bibliography search revealed a further seventeen potential studies, none of which subsequently met inclusion criteria. Therefore five studies were included for final analysis [21–25].

### Study and participant characteristics

The five studies that met final inclusion were all published in English. The publication dates ranged from 1993 to 2011, with two of the five studies conducted in the US, one in Canada and the US, one in Australia, and one in Finland. These studies enrolled a total of 1162 patients. Study and participant characteristics are outlined in Tables 1 and 2.

**Table 1:** Study characteristics

Study		Study Intervention			Control		
First author (year)	Country	Fluid (dose)	Co-interventions	Co-intervention fluid volume Mean (SD)^a^ (L)	Fluid (dose)	Co-interventions	Co-intervention fluid volume Mean (SD)^a^ (L)
Bulger et al. (2011) [21]	USA Canada	7.5% HS (250 ml)	Fluid as per local protocol	1.31 (1.07)^b^	0.9% NS (250 ml)	Fluid as per local protocol	1.16 (0.81)^b^
Jousi et al. (2010) [22]	Finland	7.5% HS (300 ml)	Ringer’s acetate and plasmafucin or hydroxyethylstarch 6%	0.8 (0.3–1.3)^c^	Ringer’s acetate and Plasmafucin or hydroxyethylstarch 6%^e^	NA	0.4 (0.2–0.5)^d^
Cooper et al. (2004) [23]	Australia	7.5% HS (250 ml)	LR or Haemacell (colloid)	Total fluid: 1.3 (0.8–2.3)^d^ Colloid: 0.5 (0.0–0.6)^d^	LR (250 ml)	LR or Haemacell (colloid)	Total Fluid: 1.3 (0.8–2.3)^d^ Colloid: 0.3 (0.0–0.5)^d^
Vassar et al. (1993a) [24]	USA	7.5% HS (250 ml)	“conventional isotonic fluids”	Pre-intervention: 1.2 (1.1) Post-intervention: 1.0 (0.8)	LR (250 ml)	“conventional isotonic fluids”	Pre-control: 1.3 (1.2) Post-control: 1.0 (0.9)
Vassar et al. (1993b) [25]	USA	7.5% HS (250 ml)	“conventional isotonic fluids”	Pre-intervention: 0.3 (0.4) Post-intervention: 1.2 (0.8)	0.9% NS (250 ml)	“conventional isotonic fluids”	Pre-control: 0.3 (0.6) Post-control: 1.1 (0.8)

Note: *Co-intervention* an intervention that is given either with the study intervention or control, *HS* hypertonic saline, *LR* Lactated Ringer’s solution, *NA* not available, *NS* normal saline

^a^Except where indicated; does not include intervention or control fluid

^b^unclear if this includes intervention/control fluid

^c^mean (range)

^d^median (IQR)

^e^There was no explicit control dose provided, but rather routine care with the does outlined in the co-intervention fluid volume column

**Table 2:** Patient Characteristics

First Author (Year)	Sample Size^a^				Inclusion	Exclusion	Age – Years Mean (SD)		Gender – Male Number (%)		Presenting SBP Mean (SD)		TBI Number (%)		Presenting GCS Mean (SD)		Blunt Trauma Number (%)	
	I	C	Total	Lost to Follow-up			I	C	I	C	I	C	I	C	I	C	I	C
Bulger et al. (2011)	256	375	631	2	1. 15 years or older 2. Prehospital SBP <70 mmHg 3. Prehospital SBP between 70 and 90 mmHg with a heart rate of 108 or less	1. Known or suspected pregnancy 2. Age less than 15 years 3. Out of hospital CPR 4. Administration of >2 L of crystalloid, colloid or blood products pre-enrollment 5. Severe hypothermia 6. Drowning, asphyxia due to hanging 7. Burns more than 20% BSA 8. Isolated penetrating head injury 9. Inability to obtain IV access. 10. Time of dispatch to study intervention more than 4 h 11. Known prisoner, interfacility transfers	36.8 (16.1)	36.2 (16.4)	205 (81%)	291 (77.4%)	54.1 (35.3)	58.1 (32.2)	None	None	10 (5)	9.8 (5)	164 (64.1%)	227 (60.4%)
Jousi et al. (2010)	17	20	37	NA	1. Patients in whom it was estimated to develop significant prehospital hypovolemia (> 1000 ml of blood loss) either from actual clinical condition or MOI. 2. MOI included multiple trauma, penetrating trauma of the head, neck, chest or abdomen, fracture of pelvic ring or femur, or a suspicion of injury of large proximal vessels of the extremities	1. Patients who received >500 ml crystalloid before assessment	37 (18)	50 (22)	12 (71%)	17 (85%)	118 (32)	125 (26)	5 (29%)	4 (20%)	12.6^c^ (3.4)	13.3^c^ (3.1)	15 (88%)	18 (90%)
Cooper et al. (2004)	114	115	229	2	1. Coma due to blunt head trauma 2. A GCS score of less than 9 3. Hypotension (SBP <100 mmHg) 4. Patients with multisystem trauma were included	1. Penetrating trauma, 2. Younger than 18 years 3. Were pregnant 4. No IV access 5. Serious pre-morbidity disease on medical identity bracelet 6. Had peripheral edema 7. Close proximity receiving hospital (“scoop and run”) 8. Had absent sinus rhythm or cardiac arrest	38 (19)	37 (19)	75 (66)	76 (66)	72^b^ (14.99)	56.2^b^ (24.51)	All participants	All participants	4.5** (1.15)	4.5** (1.15)	All participants	All participants
Vassar et al. (1993a)	50	45	95	0	1. Injured patients with systolic blood pressures less than 90 mmHg	1. Patients were asystolic or were undergoing cardiopulmonary resuscitation 2. Lacked a sinus complex on electrocardiogram 3. Appeared to be less than 18 years of age 4. Were seen more than 2 h from the time of injury 5. Were pregnant 6. Were known to have a history of seizures or a bleeding disorder 7. Appeared to have pre-existing hepatic, cardiac or renal disease as indicated by the ascites or peripheral edema. 8. Were injured as a result of a burn 9. Had a blood pressure of more than 90 mmHg by the time that IV access was established 10. Lacked IV access	31 (13)	37 (18)	NA	NA	66 (27)	72 (15)	NA	NA	8 (5)	9 (6)	41 (82%)	36 (80%)
Vassar et al. (1993b)	85	84	169	0	1. Trauma patients undergoing ground ambulance transport to the medical center if their systolic blood pressure fell to 90 mmHg or less at any time during the transport.	Same as above	32 (15)	31 (12)	NA	NA	65 (29)	64 (32)	NA	NA	12 (4)	12 (4)	49 (57.6%)	53 (63.1)

Note: *MOI* mechanism of injury, *NA* not available, *TBI* traumatic brain injury

^a^includes hypertonic and isotonic saline arms only. If it is a three arm trial, other arms (e.g., hypertonic saline and dextran, etc.) are not included

^b^Mean and standard deviation calculated based on sample size, median and interquartile range, assuming symmetrical distribution of the data

^c^ Unclear if this is presenting GCS

All studies used 7.5% hypertonic saline with a volume of 250 ml except for one study that used 300 ml [22]. Control fluids varied but all were isotonic or near isotonic fluids and used the same volume as the intervention in all cases except one [22]. These fluids included normal saline, Ringer’s Lactate and Ringer’s Acetate. Moreover, all studies provided some sort of co-intervention as part of “routine care”, which included a colloid in two studies [22, 23].

Specific inclusion criteria between studies were variable, but all required the patient to present with a blood pressure under a predetermined systolic blood pressure threshold (90 mmHg or 100 mmHg) except for one study [22]. This study used suspicion of the development of hypotension as an inclusion criteria. Likewise, exclusion was also variable, with all studies except one excluding patients in cardiac arrest [22]. One study explicitly excluded penetrating injuries [23].

Study participants were all traumatically injured and had similar ages, which ranged between a mean of 31 and 50 years. The majority of participants were male, although not all studies reported gender. Presenting systolic blood pressure for those studies using a systolic blood pressure cut-off ranged from 54 to 72 mmHg. The one study that did not use a blood pressure cut-off reported presenting systolic blood pressures of over 100 mmHg [22]. Two studies included traumatically brain injured (TBI) patients [22, 23], one did not [21], and two did not report if TBI patients were included [24, 25]. All studies reported the presenting Glasgow Coma Scale score, which ranged between eight and 13. All studies reported the number of participants with blunt trauma and this proportion ranged from 58 to 100%. Survival to hospital discharge was the only outcome that was provided by all five studies.

### Primary Outcome

**Fig. 2:**
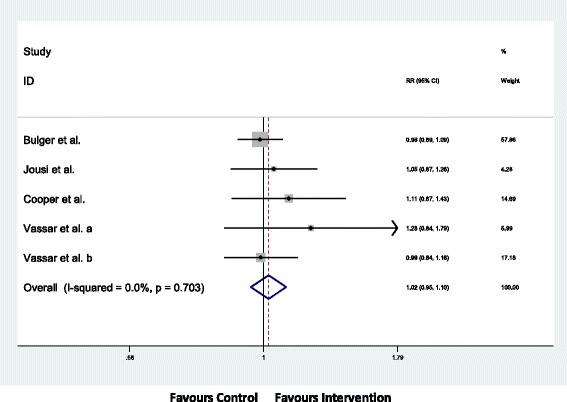
Forest Plot of Survival to Hospital Discharge using a Fixed Effect Model

The overall survival to hospital discharge was 69% in both study groups and is summarized in Table 3 and Fig. 2. The Cochran’s Q *p*-value was greater than 0.10 (*p* = 0.703), and the I^2^ was less than 40% (I^2^ = 0%) therefore a fixed effect model was specified. There was no statistically significant difference in survival to hospital discharge between the intervention and control groups when data from the five studies were pooled (RR 1.02; 95% CI 0.95, 1.10).

**Table 3:** Survival to hospital discharge

Study	Intervention		Control	
Bulger et al. (2011)	185/256	72%	276/376	73%
Jousi et al. (2010)	16/17	95%	18/20	90%
Cooper et al. (2004)	63/114	55%	57/115	50%
Vassar et al. (1993a)	30/50	60%	22/45	49%
Vassar et al. (1993b)	66/85	78%	66/84	79%

### Secondary outcomes

Secondary outcomes are summarized in Table 4. Most studies did not report any statistically significant difference in secondary outcomes with two exceptions. Bulger et al.’s study met a priori stopping rules due to a safety concern related to a statistically significant increase in 28-day mortality when patients were stratified by the post-randomization variable of blood transfusion in the first 24 h after injury [21]. In this subgroup, patients who received hypertonic saline had a 7.4% absolute increase in 28-day mortality compared to those who received normal saline (95% CI 2.5%, 12.2%) [21]. Vassar et al. reported a statistically significant greater mean change (standard deviation) in systolic blood pressure in patients receiving hypertonic saline compared to Lactated Ringer’s solution (34 mmHg ± 46 vs. 11 mmHg ± 49, *P* < 0.03) [24].

**Table 4:**
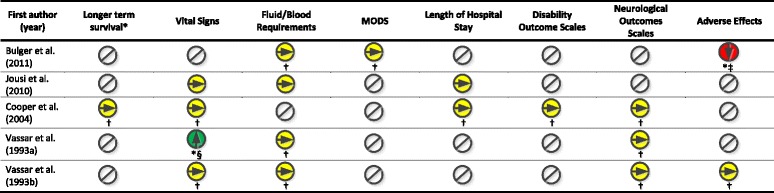
Summary of Secondary Outcomes for the intervention of hypertonic saline

Legend:

Statistically significant finding supporting intervention, Non-statistically significant finding, Statistically significant finding supporting control, Not assessed

Note: *MODS* Multiple Organ Dysfunction Score

* Defined as survival time beyond survival to hospital discharge

† Pre-specified secondary outcome for intention-to-treat analysis

‡ Patients were stratified by the post-randomization variable of blood transfusion in the first 24 h post injury. Those patients that received hypertonic saline had a 7.4% increase in 28 day mortality compared to those that received normal saline (95% CI 2.5%, 12.2%)

§ The mean change (standard deviation) in systolic blood pressure was higher in patients in the hypertonic saline group than in patients in the lactated ringers group (34 ± 46 vs 11 ± 49 mmHg, *p* < 0.03)

### Assessment of risk of bias

Overall, there was low likelihood of bias from the three a priori defined study quality criteria, although one study (Jousi et al.) did not provide adequate information to make a judgement on any of the study quality components (Table 5) [22]. Another study (Vasser et al.) reported that the hypertonic saline arm had a higher injury severity score, proportion of severe injuries to one or more body regions, and severe injury to the brain and chest compared to the normal saline control group, which may have biased the results to null as intervention patients were more severely injured [25].

**Table 5:** Risk of bias of included studies

Study (year)	Concealed allocation^a^	Blinded outcome assessors^a^	Intention to treat^a^	Actual/Planned sample size (%)	Intervention and control groups comparable
Bulger et al. (2011)	Low	Low	Low	853/3726 (23%)	High
Cooper et al. (2004)	Low	Low	Low	229/220 (104%)	High
Jousi et al. (2010)	Unclear	Unclear	Unclear	37/NA (NA)	Low
Vassar et al. (1993a)	Low	Low	Low	194/600 (32%)	High
Vassar et al. (1993b)	High	Low	Low	258/600 (43%)	Low

^a^Risk of bias rated as low, high or unclear

In addition to the aforementioned risks of bias, only one of the five studies reached their a priori defined sample size (Cooper et al.) and one study did not provide a sample size determination (Jousi et al.) [22, 23]. Bulger et al. stopped their trial early after reaching only 23% of their sample size goal [21]. Both studies by Vasser et al. were also unable to reach their sample size due to an issue with the supply of fluid [24, 25].

### Sensitivity analysis

The study by Bulger et al. contributed greater than 60% to the pooled estimate [21]. When this study was removed, the pooled effect estimate remained non-statistically significant (RR 1.07; 95% CI 0.92, 1.22). When we removed the single study where risk of bias could not be judged due to inadequate reporting, there was no significant change to the RR [22]. A random effects model yielded almost identical results to the fixed effect model (RR 1.01; 95% CI 0.94, 1.09).

### Publication bias

**Fig. 3:**
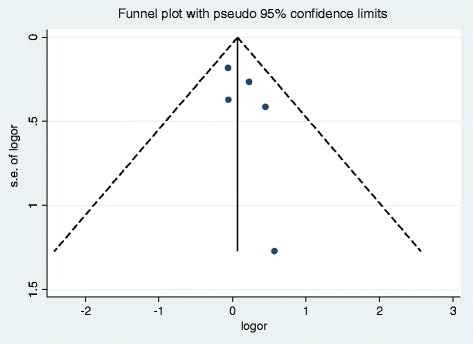
Funnel plot of studies included for meta-analysis (*n* = 5)

Small study effect was assessed using both the Egger’s test and Begg test. Neither test suggested untoward influence from small studies in this review (*p* = 0.62 and *p* = 0.87 respectively). Moreover, an analysis of the funnel plot did not suggest asymmetry from small study effects (Fig. 3).

## Discussion

This meta-analysis of 1162 hypotensive prehospital patients from five studies comparing prehospital administration of 7.5% hypertonic saline compared to isotonic fluid in hypotensive patients did not demonstrate a statistically significant change in survival to hospital discharge. There were no consistent significant differences in secondary outcomes.

These findings align with two previous systematic reviews and meta-analyses of hypertonic saline administration to trauma patients, although these reviews included studies in both the prehospital and hospital setting.

The results of our systematic review suggest that there is no evidence of benefit to the prehospital application of hypertonic saline for hypotensive injured patients. It is important, however, to consider how the included patients and the fluids may have contributed to these findings.

Patients presenting to EMS may be categorized broadly on a spectrum from life-threatening injury to non-life threatening injury. The former patients may die regardless of EMS interventions, where the latter patients may survive regardless of EMS interventions. In the middle of this spectrum are patients with life-threatening injuries amenable to appropriate EMS therapy. It is these patients that have the potential to benefit from an effective intervention, but may be a small number within the larger population of EMS patients. Identifying these patients in the very narrow assessment time that is afforded to paramedics is difficult. Indeed this was the challenge outlined by Brasel and colleagues (2008) when designing the ROC trial reported by Bulger et al. [21, 26]. They changed the inclusion from a broad approach of patients presenting with a SBP less than or equal to 90 mmHg, to a SBP less than or equal to 70 mmHg, or a patient with a SBP less than or equal to 90 mmHg and a heart rate greater than or equal to 108 beats per minute. The rationale for this change was to identify a population that was more likely to receive greater than 10 units of packed red blood cells in the first 24 h (i.e., not “mildly injured” patients), based on a previous study in 2008 [26, 29]. All studies included in this review, except Bulger et al. used a broad systolic blood pressure categorization [21]. This may mean that the group of patients that would potentially benefit from hypertonic saline are much smaller than the reported sample sizes, resulting in a bias of the effect estimate towards null. Finally, it is unclear what impact the administration of hypertonic saline might have in hypotensive patients with traumatic brain injury [30, 31].

The fluid that the patient received, as part of the study or as part of routine care, either before or after enrolment in the study is important. It has been postulated that receiving isotonic fluid either before or after hypertonic saline may negate the beneficial effects of hypertonicity [6]. All studies included in this review permitted the use of isotonic fluid before and/or after administration of the study fluid. It is unknown what magnitude of effect this may have had on outcome, but if isotonic fluids negate the effect of hypertonicity, then this would decrease the potential effect of hypertonic saline and move the effect estimate towards null. In addition, the volume of hypertonic saline provided to intervention groups was between 250 ml (4 out of 5 studies) and 300 ml. This dose has also been criticized as it may be too low compared to the weight adjusted dose used in previous animal studies [6]. While 4 ml/kg has been reported as the hypertonic saline dose used in the original animal studies, in the included studies an 80 kg (175 lb) patient would have received an approximately 3 ml/kg dose and a 50 kg (110 lb) patient a 5 ml/kg dose. Conducting a subgroup analysis based on patient weight was not possible given the available data [6, 32]. Since the majority of patients in these trials were male (range 66% to 85%), it is possible that many participants were systematically under dosed hypertonic saline compared to the original animal studies. This would also contribute towards decreasing the potential effect of hypertonic saline and move the effect estimate towards null.

It must be noted that the Bulger et al. trial was stopped early due to futility in the presence of a possible safety concern [21]. The authors suggest that the “mortality” effect in patients that received blood transfusion in the first 24 h post injury may be caused by a shift to earlier mortality in those patients receiving hypertonic saline, although this could not be statistically demonstrated. They further suggest that this shift to earlier mortality may occur because of increased bleeding in the hypertonic saline group, and possibly a change in physician behaviour that delayed the administration of transfusions [21]. Other authors have disputed that increased bleeding is a plausible explanation based on the reported hemoglobin levels, and have suggested that it was indeed a failure on the part of the first-receiver physician to recognize a shock state in the face of non-shock level systolic blood pressure and cutaneous hyperemia that occur with the use of hypertonic saline [6, 32]. Other authors have postulated that it is the creation of subgroups using a post-randomization variable (number of blood transfusions) that has introduced a “collider bias” in the study analysis [33]. The authors suggest that the “increased mortality” in the hypertonic saline /no transfusion group is a systematic bias rather than a clinical phenomenon [33].

### Limitations

The homogenous nature of the intervention allowed for pooling of results, but other concentrations of hypertonic saline (e.g., 3%, 5%, etc.) [34], doses, or weight-based dosing regimens were not assessed. It is possible that a single 250 ml dose of 7.5% hypertonic saline may not be sufficient to evaluate the clinical effectiveness of hypertonic saline.

No trials except Cooper et al. (2004) reached planned sample size [23]. However, some authors have also criticized the Cooper et al. study for being underpowered [1]. All trials had low power to exclude clinically important differences, especially for secondary outcomes. The 95% confidence interval of the pooled estimate spanned a potential reduction in survival of 5% to an increase in survival of 10%. While not excessively wide, this estimate would have been more precise had studies reached their planned sample sizes and includes effects that could be clinically important.

Every effort was made to conduct thorough searches of major research databases, and be inclusive in the title and abstract review, however it is still possible that trials were missed. This is especially true for newer studies that may not have been published, or only recently published in full text and not yet indexed. Hand searching of conferences was not performed. Finally, all studies included injured patients located in civilian EMS settings, and did not include patients with other causes of hypotension or military far-forward, tactical or wilderness EMS settings where the theoretical benefits of hypertonic saline may be more readily demonstrated.

## Conclusions

This systematic review and meta-analysis demonstrated no significant difference in important clinical outcomes for hypotensive injured patients administered hypertonic saline compared to isotonic fluid in the prehospital setting. Hypertonic saline cannot be recommended for use in prehospital clinical practice for the management of hypotensive injured patients based on the available data. Given the discordance between pre-clinical and clinical trials, further evaluation is warranted from studies that create pragmatic inclusion criteria to enrol patients most likely to benefit from hypertonic saline, and to use weight based doses and differing concentrations with routine care that maintains hypertonicity for longer periods of time.

## Abbreviations

EMS: Emergency medical services
MODS: Multiple organ dysfunction score
RCT: Randomized controlled trial
TBI: Traumatically brain injured
